# Quantum ratchet in two-dimensional semiconductors with Rashba spin-orbit interaction

**DOI:** 10.1038/srep07872

**Published:** 2015-01-19

**Authors:** Yee Sin Ang, Zhongshui Ma, Chao Zhang

**Affiliations:** 1 School of Physics, University of Wollongong, New South Wales 2522, Australia; 2 School of Physics, Peking University, Beijing 100871, China; 3 Collaborative Innovation Center of Quantum Matter, Beijing, 100871, China and; 4 Cooperative Innovation Center on Terahhertz Science and Technology, Chengdu, China

## Abstract

Ratchet is a device that produces direct current of particles when driven by an unbiased force. We demonstrate a simple scattering quantum ratchet based on an asymmetrical quantum tunneling effect in two-dimensional electron gas with Rashba spin-orbit interaction (R2DEG). We consider the tunneling of electrons across a square potential barrier sandwiched by interface scattering potentials of unequal strengths on its either sides. It is found that while the intra-spin tunneling probabilities remain unchanged, the inter-spin-subband tunneling probabilities of electrons crossing the barrier in one direction is unequal to that of the opposite direction. Hence, when the system is driven by an unbiased periodic force, a directional flow of electron current is generated. The scattering quantum ratchet in R2DEG is conceptually simple and is capable of converting a.c. driving force into a rectified current without the need of additional symmetry breaking mechanism or external magnetic field.

## Introduction

Ratchet is a device that produces direct current of particles when driven by an unbiased force^1^,^2^. In technological applications, ratchets are particularly useful in nano-electronics as they can be utilized as miniature current rectifiers, switches or refrigerators^3^,^4^. Ratchet plays an important role in many biological processes such as the intracellular transport of proteins and ATP hydrolysis^5^,^6^. To create directed motion of particles, a ratchet structure must possess some form of spatial or temporal symmetry breaking^7^. For example, the thermal diffusion of particles can be ‘chopped’ by a time-modulated asymmetrical potential barrier and this leads to a directed motion of particles^8^,^9^. Alternatively, net flow of particles across asymmetrical potential barrier can also be driven by dichotomous Markov noise^10^,^11^. Such devices belongs to the class of *classical Brownian ratchets* since the ratchet current originates from the classical Brownian diffusion of particles. When the quantum tunneling of particles across the asymmetrical confining barrier is taken into account, the ratchet current is significantly enhanced and it exhibits a directional reversal dependent on the temperature and the period of the external fields^12^,^13^. The quantum ratchet effect has been experimentally demonstrated in the transport of electrons through asymmetric conducting channels in GaAs/AlGaAs heterostructure^14^. Quantum ratchet motion of Rubidium atoms has also been realized via time-modulated optical lattice^15^. Alternatively, transport asymmetry can be generated in a two-dimensional electronic system with layer asymmetry in the presence of an in-plane magnetic field. Drexler et al has elegantly demonstrated this *magnetic quantum ratchet effect* in semihydrogenated graphene where the layer symmetry is broken by the selective attachment of hydrogen adatoms to only one surface of the graphene layer^16^. In such structure, the in-plane magnetic field is coupled to the terahertz (THz) excitation of the electrons to produce out-of-plane Lorentz forces. The direction of the Lorentz forces are dependent on the in-plane directions of the THz-driven electrons. Electrons that are pushed towards the adatoms experience enhanced scattering and this leads to a directed flow of electrons.

In this paper, we describe a *scattering quantum electron ratchet* in two-dimensional electron gas with Rashba spin-orbit interaction (R2DEG)^17^,^18^,^19^. It has been shown that the Rashba spin-orbit coupling can results in zero field Hall current^20^, specular Andreev reflection^21^, and chiral tunneling^22^ in semiconductors. It can also give rise to the low frequency conductance resonance in graphene^23^. In the present problem, the ratchet current originates from the asymmetrical tunneling of electrons across a potential barrier sandwiched by two interface scattering potentials of unequal strengths. We found that although the tunneling probabilities of the *same-spin-subband* transmission is symmetrical for electrons tunneling across the junction in both directions, this symmetry is broken in the case of the *inter-spin-subband* tunneling process. When the tunnel junction is periodically driven, the left-going and the right-going tunneling currents are unequal. Such asymmetrical tunneling of electrons in R2DEG leads to a net transfer of electrons across the tunnel junction driven by a sinusoidal bias voltage.

### Model and Formalism

In order to investigate the transport properties in a R2DEG tunneling junction, we first review the electronic properties of R2DEG shortly. In a quantum well structure, two-dimensionally confined electrons can undergo spontaneous lifting of the spin-degeneracy if the confining potential is asymmetric. Such effect is equivalent to the relativistic case of electron moving through a surface with inhomogeneous electric field. In the rest frame of the electrons, the electric field is relativistically equivalent to a magnetic field. This effectively generates finite spin-orbit interaction and energetically separates the electron gas into two populations of different spin chirality. Spin-orbit-interaction of this form is known is the Rashba spin-orbit interaction (RSOI)^17^. The RSOI manifests itself as a left-and right-shifting of the ‘free’ electron parabolic bands in phase-space and the degree of the splitting is characterized by a Rashba coupling parameter *λ*^18^,^19^.

**Figure 1:**
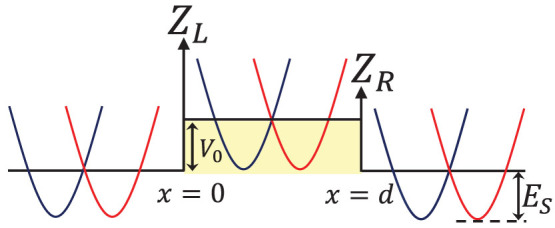
Model structure of the R2DEG tunneling junction in the presence of interface scattering potentials. Electrons incident from the left hand side of the potential barrier. The square potential barrier height is *V*_0_. *Z_L_* and *Z_R_* represents the left and the right interface scattering potentials respectively.

Although the tunneling problems in R2DEG has previously been studied^24^,^25^,^26^,^27^,^28^,^29^,^30^,^31^,^32^, it is not clear whether the presence of an interface scattering potentials can play a role in the electron transport of this system. This is the main objective of this work. In order to study the effect of the interface scattering potential on the spin-polarized transport, we model a square potential barrier *V* (*x*) in the width *d*. The inhomogeneities for the left and right interface scatterings are described by introducing two delta interface potentials of the strengths *Z_L_*_/*R*_, i.e. *V*(*x*) = (Θ(*x*)−Θ(*x*−*d*)) *V*_0_+*Z_L_δ*(*x*)+*Z_R_δ*(*x*−*d*) [see Figure 1(a)]. In practice, the interface scattering potential can be achieved by applying thin strips of electrostatically-gated electrodes to the R2DEG confined in a GaAs/AlGaAs heterostructure, and the square barrier height *V*_0_ can be controlled by gate voltage on the scattering region of the tunneling structure. The Hamiltonian of infinite R2DEG is given as^17^


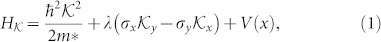
where 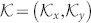 is the wavevector, m* is the electron effective mass, *σ*_x_ and *σ*_y_ are the Pauli spin matrices and λ is the Rashba coupling parameter. In our model, we shall ignore the interaction between R2DEG and phonons^33^. This equation can be written in a form *h_k_* = *k*^2^+2(*σ_x_k_y_*−*σ_y_k_x_*) which introduces only the following dimensionless quantities: 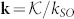, 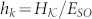 and *v*_0_ = *V*_0_/*E*_SO_ with *k*_SO_ = *mλ*/ħ^2^ and 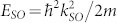. The eigenvalue of the reduced Hamiltonian *h_k_* without the potential barrier (i.e. *v*_0_ = 0) is *ε_s_* = *k*^2^+2*sk*, where *s* = ±1 represents the chirality of the spin-subband. The wavevector of state *s* = +1 is given as 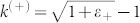. There are two situations corresponding to the state *s* = −1. When ε_−_>0, there is only one wavevector, 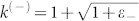. However, when −1<*ε*_−_<0 there are two wavevectors 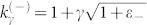 where *γ* = ±1. The index *γ* denotes the outer (*γ* = +1) and the inner (*γ* = −1) Fermi circle of the *s* = −1 subband. For the eigenvalue *ε_s_*>0, the eigenstate of Eq. (1) is given as 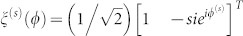 where *T* stands for transpose and 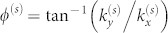 is the azimuthal angle of the wavevectors 
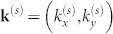
. For *s* = −1 state with energy −1<*ε*_−_<0, the eigenstate can be expressed in the form of 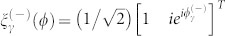, where 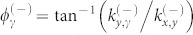 is the azimuthal angle of the wavevectors 
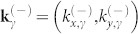
. Corresponding to these wavevectors, the propagation of the eigenstates manifests in different transmittal characteristics. To see this, we first look at the group velocity of the electrons. When *ε*_s_>0, the group velocity in *x*-direction, defined as 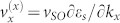, is given as 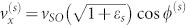, while 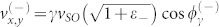 for −1<*ε*_−_<0, where *v_SO_* = *E_SO_*/ħ. Because the sign of 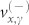 is determined by γ, the group velocity 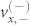 is negative. In this case, the wavevector is anti-parallel with the direction of motion. This infers a hole-like characteristic for the electrons residing in the *s* = −1 and *γ* = −1 branch.

Now we apply these discussions to our system. For an incident in the left in eigenstate 
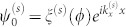
, the *s*→*s*′, with *s*′ = ±1, reflection process from the left interface of the barrier layer is in the rate of 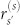, and can be written as 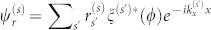. The wavefunction in the incident side is hence 
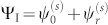
. In the barrier region, the wave-function is 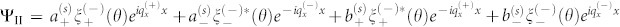, where we denote 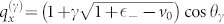 for the wavefunction in the barrier layer. The wavefunction in the drain is given by 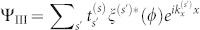, where the coefficients 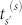 represent the strengths of the *s*→*s*′ transmission. In these wavefunctions, the conserved factor 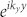 has been omitted for simplicity. The wavevector 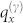 in the barrier layer is real for *v*_0_−1<*ε*_−_<0 and evanescent for *ε*_−_<*v*_0_−1. The transmission and reflection coefficients can be readily solved from the conservation condition of the *y*-component of the wavevector and the matching of the wavefunctions at different regions via the boundary conditions: Ψ_I(II)_ = Ψ_II(III)_ and ∂Ψ_I(II)_/∂*x*−∂Ψ_II(III)_/∂*x* = (2*mZ_L_*_(*R*)_/ħ)Ψ_I(II)_ at the boundaries *x* = 0 (*x* = *d*). Finally, the transmission and reflection probabilities are given as 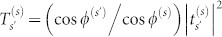 and 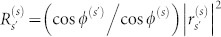.

### Tunneling without the interface scattering potential

**Figure 2:**
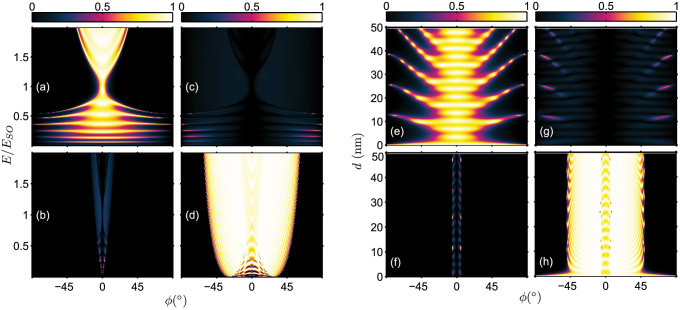
Energy spectrum and the barrier width dependence of the transmission probabilities in the absence of interface scattering potentials. Energy dependence of (a) 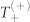; (b) 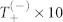; (c) 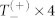; and (d) 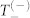. Barrier width dependence of (e) 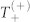; (f) 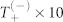; (g) 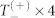; and (h) 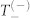. The tunneling junction parameters are *V*_0_ = *E_SO_* and *k_SO_* = 1.3 × 10^9^ m^−1^. For (a)–(d), *d* = 20 nm and for (e)–(h), *E* = 0.5*E_SO_*.

For the case without interface scattering potential at the *x* = 0 and *d* interfaces, the energy dependence of the transmission probabilities is shown in Figure 2. The transmission probabilities for the same-branch process+→+and the inter-branch process+→ − are shown in Figure 2(a) and Figure 2(c), respectively. In comparison with Figure 2(c), Figure 2(a) shows that the same-branch transmission is much stronger than the inter-branch transmission. Similar, for *s* = −1 incident state, transmission via the process − → − is also much stronger than that of the process − →+[see Figure 2(b) and Figure 2(d)]. For the − → − process, the probability oscillations occurs for both under-and over-barrier incident energy [Figure 2(d)]. The barrier width dependence of the transmission probabilities is shown in Figure 2(e)–(h) and Figure 2(e) and 2(g) for *s* = +1 and *s* = −1 incident states, respectively. For *s* = +1 incident states, both+→+and+→ − transmissions are rich in features and extends over a very large angular range. Oscillation of the transmission probabilities is particularly obvious in the small incident angle regime of the+→+process. For the *s* = −1 incident states, the − →+transmission is, however, confined only in a relatively smaller angular range [Figure 2(f) and 2(h)]. For − →+process, transmission can only occur via very small angle of incidence regardless the barrier width because the Fermi radius of the *s* = +1 transmitted state is much smaller than that of the *s* = −1 incident states.

### Tunneling in the presence of symmetrical interface scattering potentials

**Figure 3:**
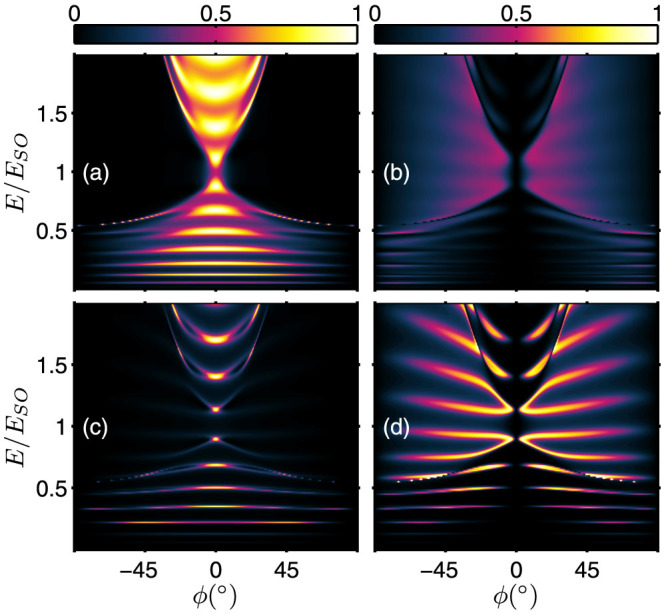
Energy spectrum of the transmission probabilities in the presence of symmetrical interface scattering potential *Z_L_* = *Z_R_*. *Z_L_* = *Z_R_* = 0.5: (a) 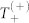; (b) 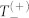; and *Z_L_* = *Z_R_* = 1.5: (c) 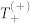; and (d) 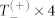. The tunneling junction parameters are *d* = 20 nm, *V*_0_ = *E_SO_* and *k_SO_* = 1.3 × 10^9^ m^−1^.

**Figure 4:**
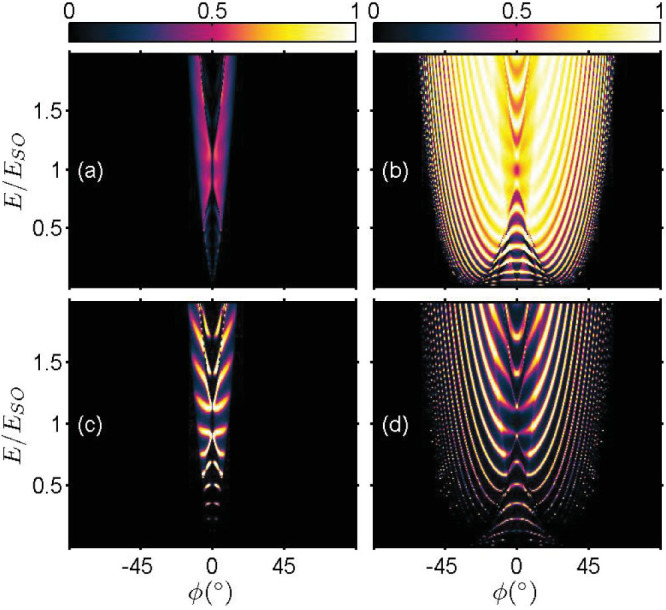
Energy spectrum of the transmission probabilities in the presence of symmetrical interface scattering potential *Z_L_* = *Z_R_*. *Z_L_* = *Z_R_* = 0.5: (a) 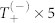; (b) 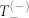; and *Z_L_* = *Z_R_* = 1.5: (c) 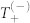; and (d) 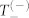. (The junction tunneling parameters are the same as Fig. 4)

We now consider the case when *symmetrical* interface scattering potentials are present, i.e. *Z_L_* = *Z_R_*. The energy dependence of the transmission probabilities for different strength of interface scattering potentials is shown in Figure 3 and Figure 4 respectively for *s* = +1 and *s* = −1 incident states. As an anticipatory result, electron tunneling is, in general, suppressed by the interface scattering potentials. However, there is an exception for the inter-branch transmissions of +→ − and − →+. For the 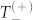 transmission, direct comparison of Figure 2(c) with Figures 3(b) and 3(d) shows that a stronger interface scattering potential actually produces narrow strips of enhanced +→ − inter-branch tunneling. Similarly, comparison of Figure 2(b) with Figures 4(a) and 4(c) also indicates the transmission 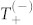 is enhanced by the presence of a stronger interface scattering potential.

### Tunneling in the presence of asymmetrical interface scattering potentials

**Figure 5:**
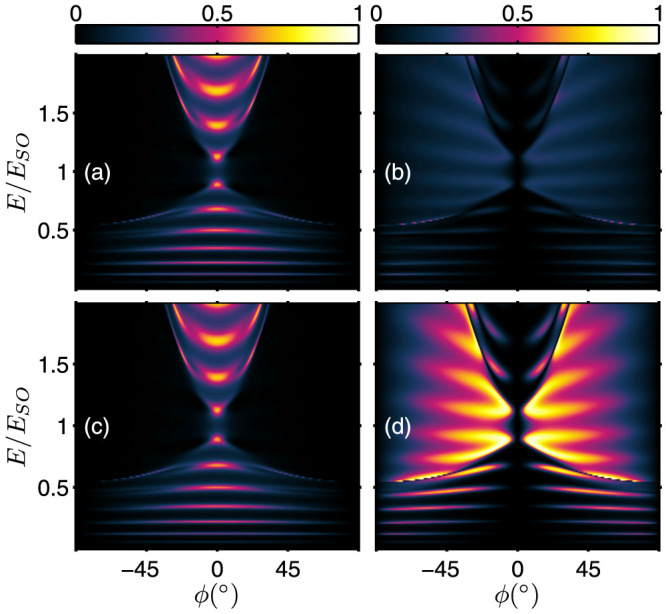
Energy spectrum of the transmission probabilities in the presence of asymmetrical interface scattering potential, *Z_L_* ≠ *Z_R_*. (a) 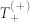; and (b) 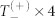 for *Z_L_* = 0.5 and *Z_R_* = 1.5; (c) 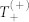; and (d) 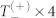 for *Z_L_* = 1.5 and *Z_R_* = 0.5. (The junction tunneling parameters are the same as Figure 4)

**Figure 6:**
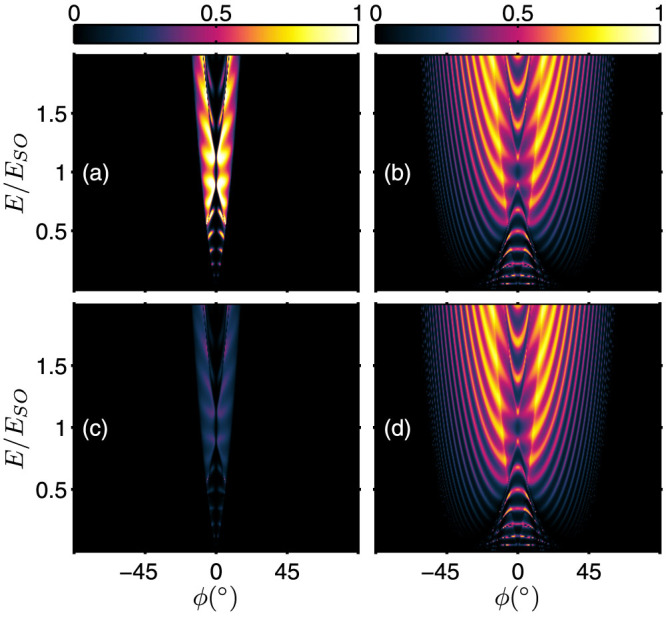
Energy spectrum of the transmission probabilities in the presence of asymmetrical interface scattering potential, *Z_L_* ≠ *Z_R_*. (a) 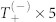; and (b) 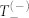 for *Z_L_* = 0.5 and *Z_R_* = 1.5; (c) 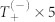; and (d) 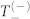 for *Z_L_* = 1.5 and *Z_R_* = 0.5. (The junction tunneling parameters are the same as Figure 4)

We now investigate the case when the interface scattering potentials are asymmetrical for the left and right boundaries, i.e. *Z_L_* ≠ *Z_R_*. The transmission spectra of the *s* = +1 incident states is shown in Figure 5. In Figure 5(a) and 5(b), the interface scattering potentials are *Z_L_* = 0.5 and *Z_R_* = 1.5, while in Figure 5(c)–(d), the interface scattering potential strengths are swapped i.e. *Z_L_* = 1.5 and *Z_R_* = 0.5. By comparing Figure 5(a) and Figure 5(c), we immediately see that the 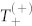 same-branch transmission is unaltered when interchanging 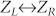. On the other hand, the results of Figure 5(b) and Figure 5(d) show distinctly that the 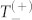 inter-branch transmission is *enhanced* when the interface scattering potentials are swapped from *Z_L_*<*Z_R_* to *Z_L_*>*Z_R_*. The phenomenon also occurs when the incident state is in the *s* = −1 branch as shown in Figure 6. The 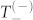 same-branch transmission remains unchanged when *Z_L_* and *Z_R_* are interchanged [Figure 6(b) and Figure 6(d)] while the 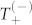 inter-branch transmission is *suppressed* when the potentials are interchanged from *Z_L_*<*Z_R_* [Figure 6(a)] to *Z_L_*>*Z_R_* [Figure 6(c)]. Therefore, different from the unaltered same-branch transmission, the inter-branch transmission is altered when 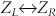. The electron tunneling becomes asymmetrical when *Z_L_* ≠ *Z_R_*.

### Scattering quantum ratchet in a R2DEG tunneling junction

In above, we have seen that the electron tunneling can be asymmetrical in the presence of asymmetrical interface scattering potentials. We can use this property of R2DEG tunneling junction to obtain a net transfer of spin-polarized electrons across the barrier via a alternating bias voltage. In this sense, the potential barrier acts as a *quantum ratchet*.

**Figure 7:**
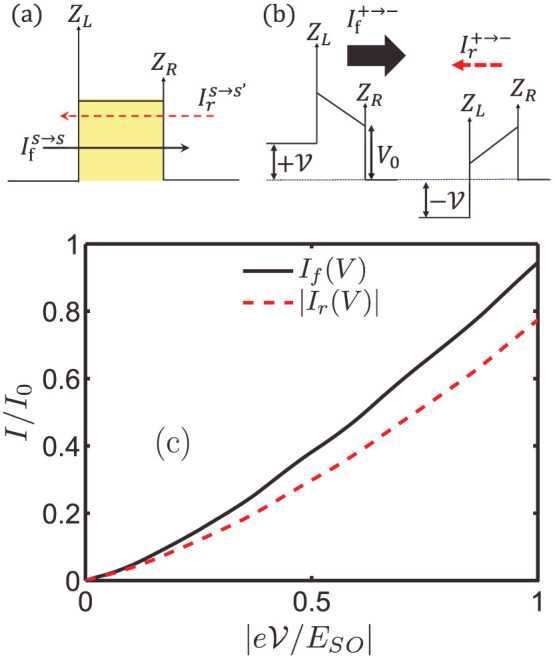
Scattering quantum ratchet in R2DEG. (a) Schematic drawing of the right-going current 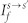 and 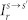 ; (b) 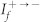 and 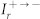 becomes unequal in the presence of asymmetrical *Z_L_* and *Z_R_*; (c) the current-voltage characteristic of the R2DEG tunnel junction. (*E_F_* = 0.5*E_SO_*, *d* = 20 nm, *V*_0_ = *E_SO_*, *Z_L_* = 1.5 and *Z_R_* = 0.5)

To see how the R2DEG tunnel junction with asymmetrical interface scattering potential can work as a quantum ratchet when it is driven sinusoidally, we apply an a.c. bias voltage to the R2DEG tunnel junction with asymmetrical interface scattering potentials (*Z_L_*>*Z_R_*) [Figure 7(a)]. In the first half of the a.c. period, a forward current *I_f_* is driven from the left to the right of the barrier and the right-moving *I_f_* ‘sees’ the left interface ‘obstacle’ *Z_L_* first and then the right *Z_R_*. In the second half period of the a.c. cycle, the current is reversed and *I_r_* is driven from the right to the left of the barrier. Due to the directional reversal, the relative order of the interface scattering potentials as ‘seen’ by *I_r_* is reserved, i.e. it ‘sees’ *Z_R_* first and then *Z_L_*. The previous calculations told us that the tunneling probabilities 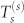 remains the same when 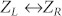. Accordingly, the same-spin tunneling process (*s*→*s*) is not affected by the interchanging of *Z_L_* and *Z_R_*. In this case, *I_f_*−*I_r_* = 0 and no net charge is transferred. However, for the opposite spin tunneling process (*s*→−*s*), 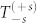 no longer remains constant when the interface scattering potentials 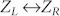 is interchanged. As a result, *I_f_*−*I_r_* ≠ 0 and a net transfer of electrons through the tunnel junction is produced [Figure 7(b)]. Since the ratchet current has its root from the unequal scattering strengths of the interface scattering potentials, the tunnel junction can be regarded as a *scattering quantum ratchet*.

We now look at the 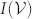 characteristic of the junction under a d.c. bias 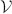 first. The charge current is given as:


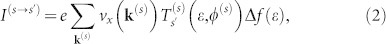
where Δf(*ε*) = *f*(*ε*−*ε_F_*−*ev*)−*f*(*ε*−*ε_F_*) with *ε_F_ = E_F_/E_SO_* and 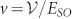. At zero temperature, we obtain:


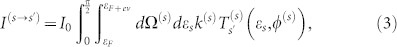
where *I*_0_ = *ek_SO_E_SO_L*^2^/(2π^2^ħ) and Ω*^(s)^* = sin *φ^(s)^*. When the LHS of the tunnel junction is raised by 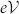
(i.e. ‘forward-bias’), the right-moving current takes the same form as Eq. (3) with the transmission 
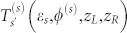
. When the RHS of the junction is raised by 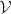
(i.e. ‘reverse-bias’), the left-moving current, 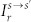, has the same form as that of the right-moving current except that *Z_L_* and *Z_R_* are interchanged. Finally, the total forward-biased and reserve-biased currents are: 
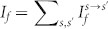
 and 
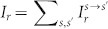
 respectively. We plot the current-voltage characteristics in Figure 7(c). For easy comparison, the absolute value of the negative-valued *I_r_* is taken. We see that *I_f_* and *I_r_* is unequal. The magnitude of If is about 20% larger than that of *I_r_* at a bias voltage of 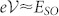.

**Figure 8:**
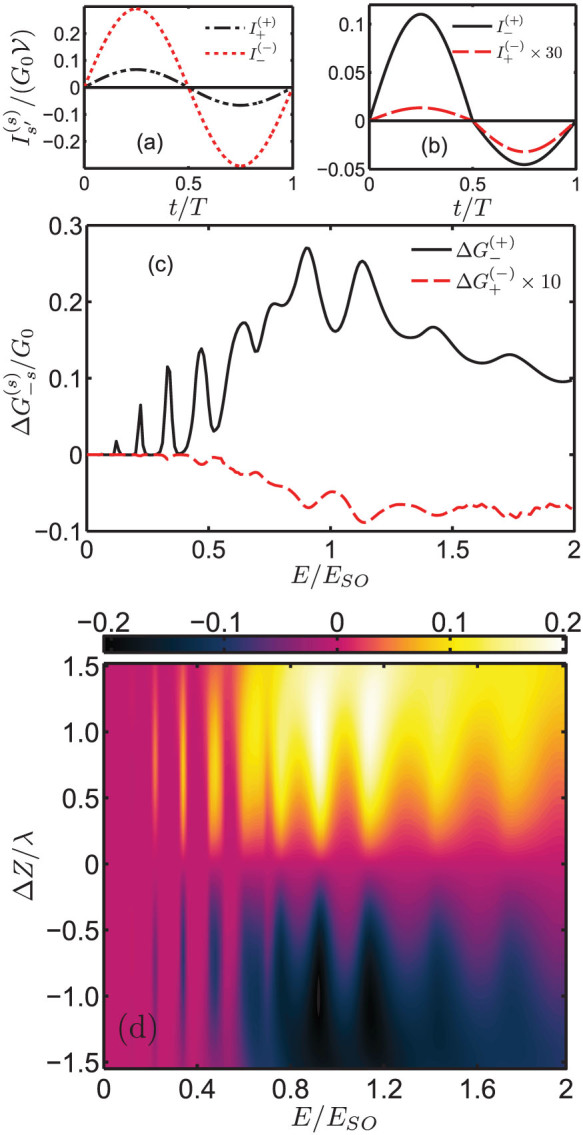
Tunneling conductance and ratchet conductance under an a.c. bias. (a) Time profile of 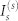 and; (b) 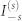 under small a.c. bias voltage. (c) Fermi level dependence of 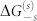 (*E_F_* = 0.5*E_SO_*, *d* = 20 nm, *V*_0_ = *E_SO_*, *Z_L_* = 1.5 and *Z_R_* = 0.5). (d) Δ*Z* and Fermi level dependence of the ratchet conductance Δ*G_tot_*. When the asymmetry of the interface potential is swapped from *Z_L_*>*Z_R_* to *Z_L_*<*Z_R_*, the ratchet current reverses its direction as signified by Δ*G_tot_*<0. When the Fermi level is very large, the ratchet current is suppressed.

We now consider the junction being driven by a symmetrical a.c. bias voltage in the form of 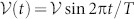 where *T* is the a.c. period. Assuming that the magnitude of 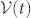 is small, only states at the Fermi level can contribute to the current. In the first half of the cycle, a current is driven rightwards across the junction, and the differential conductance, 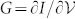, is given as


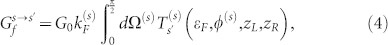
where *G_0_* = *e*^2^*k_SO_E_SO_L*^2^/(2π^2^ħ). In the second cycle, the conductance is in the same form as Eq.(4) except that 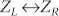. In Figure 8(a) and 8(b), we plot the time profile of the *s*→*s*′ tunneling current 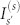. For *s*→*s* tunneling process, 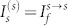 for the first-half cycle and 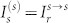 for the second-half cycle. Since 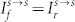 regardless the interchanging 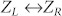, the same-spin tunneling current is a symmetrical oscillation without a net charge transfer [Figure 8(a)]. For the opposite-spin current, the first-half and the second-half cycle tunneling current are 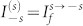 and 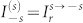 respectively. Because of 
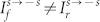
 as 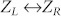, the inter-spin tunneling current oscillates asymmetrically [Figure 8(b)]. Although 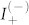 is moving in the opposite direction to 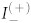, it is too small to off-set 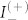. The net result is the formation of a ratchet current across the tunnel junction. The magnitude of the *s*→−*s* ratchet current is proportional to the difference 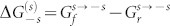
[Figure 8(c)]. 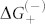 is much smaller than 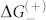 because the Fermi circle of the *s* = −1 incident states is much larger than that of the *s* = +1 transmitted states; many of the incident states are ‘squeezed’ outside of the *s* = +1 Fermi circle of the transmitted states and become evanescent. 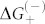 becomes noticeably larger for *E_F_*>*E_SO_* when the mismatch of the incident and the transmitted state Fermi circles becomes less severe. Such mismatch does not occur in 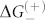 since the incident *s* = +1 Fermi circle can always fit into a transmitted state in the larger *s* = −1 Fermi circle. Rapid oscillation of 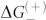 occurs at *E_F_*<0.8*E_SO_*.

The magnitude of the total ratchet current is determined by the *ratchet conductance* 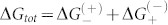. In Figure 8(d), we show the Δ*Z* and the Fermi level dependence of the ratchet conductance where *Z_L_* = *Z*_0_−Δ*Z* and *Z_R_* = *Z*_0_+Δ*Z* with *Z*_0_ = 1.5*λ*. A similar conductance oscillation is also present since Δ*G_tot_* is dominated by 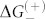. Furthermore, the direction of the ratchet current reverses when Δ*Z* changes its sign. This allows the direction of the ratchet current to be manipulated by interchanging the scattering strengths of the LHS and RHS interface scattering potentials. It should be emphasized that the results of Eq. (4) provides a qualitative picture of the quantum ratchet. This quasi-static treatment is only valid when the amplitude and the frequency of the a.c. driving field are small. We used this simple treatment to illustrate that it is possible to create a ratchet effect in R2DEG junction due to the asymmetrical *s*→−*s* transmission behaviour. For a more general a.c. driving force, time-dependent methods, e.g. Floquet methods^34^ and Keldysh non-equilibrium Green function technique^35^, should be utilized. The main error of the quasi-static treatment is that the quantum states in the leads are assumed to be independence of the electron-ac field-coupling. This effect can be large if the amplitude of the ac-field is large.

We now briefly compare our system with a similar tunneling junction of metal/R2DEG/metal^33^. In such junction, a magnetic *δ*-potential is formed at both of the metal/R2DEG interfaces due to the abrupt discontinuity of the Rashba coupling strength. They observed an adjustable spin polarized transmission of up to 10% spin-polarization. Interestingly, spin-dependent transmission is also present in our system albeit the fact that there is no Rashba coupling strength discontinuity in our case. Since the spin-dependent transmission is one of the key features that results in the scattering quantum ratchet effect, we expect the ratchet effect to be affected by the presence of such *δ* interface potential in a R2DEG tunneling junction of unequal Rashba coupling strengths at different tunneling regions.

Finally, we emphasize that the scattering quantum ratchet cannot occur in a ‘normal’ 2DEG without the Rashba spin-orbit coupling. We solve the transmission probability *T* through a potential barrier of *V*(*x*) = (Θ(*x*)−Θ(*x*−*d*)) *V*_0_+*Z_L_δ*(*x*)+*Z_R_δ*(*x*−*d*). It is found that *T* can be written as:


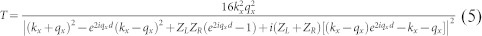
where 
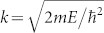
, 
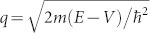
, *k_x_* = *k cos φ* and *q_x_* = *q cos θ*. *φ* is the azimuthal angle of the wavevector k, and θ can be determined by the wavevector conservation condition *k* sin *φ = q* sin *θ*. It is immediately obvious that, regardless *E>V* or *E<V*, the interchanging of 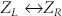 has no effect on *T*. Therefore, the scattering quantum ratchet described here *cannot occur* in normal 2DEG.

## Discussion

We have studied the electron tunneling ratchet phenomenon in R2DEG through a square potential barrier with asymmetrical interface scattering potentials in R2DEG. We found that probabilities for the same-spin tunneling (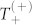
and 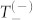) remain unchanged while probabilities for the inter-spin tunneling (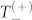
and 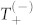) becomes unequal when the left and the right interface scattering potentials are interchanged. We then discussed a strategy to construct a *scattering quantum ratchet* based on these asymmetrical tunneling behaviors. The scattering quantum ratchet in R2DEG is conceptually simple and is capable of converting a.c. driving force into a rectified current without the need of asymmetrical transport channels^14^,^36^,^37^, optical tweezers^8^,^9^,^15^,^38^, quantum dots^39^, THz excitation and strong magnetic fields^16^,^40^. Since the scattering quantum ratchet involves only one square potential barrier, the physical dimension of such device can be greatly reduced.

## Methods

The main results of this work, i.e. the transmission probabilities 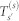 are derived using the standard wavefunction matching at the boundaries of the potential barriers. This is outlined in detail in the **Model and Formalism Section.**

## Author Contributions

Y.S.A., Z.M. and C.Z. initiated the idea and performed the analysis. Y.S.A. performed the numerical calculation. All authors co-wrote and revised the manuscript.
